# Dissecting Transcription Factor-Target Interaction in Bovine Coronavirus Infection

**DOI:** 10.3390/microorganisms8091323

**Published:** 2020-08-30

**Authors:** Olanrewaju B. Morenikeji, Ellis Strutton, Madeleine Wallace, Kahleel Bernard, Elaine Yip, Bolaji N. Thomas

**Affiliations:** 1 Department of Biology, Hamilton College, Clinton, NY 13323, USA; omorenik@hamilton.edu (O.B.M.); estrutto@hamilton.edu (E.S.); mwallace@hamilton.edu (M.W.); kbernard@hamilton.edu (K.B.); eyip@hamilton.edu (E.Y.); 2 Department of Biomedical Sciences, College of Health Sciences and Technology, Rochester Institute of Technology, Rochester, NY 14623, USA

**Keywords:** transcription factors, prediction, markers, regulation, cattle, disease

## Abstract

Coronaviruses are RNA viruses that cause significant disease within many species, including cattle. Bovine coronavirus (BCoV) infects cattle and wild ruminants, both as a respiratory and enteric pathogen, and possesses a significant economic threat to the cattle industry. Transcription factors are proteins that activate or inhibit transcription through DNA binding and have become new targets for disease therapies. This study utilized in silico tools to identify potential transcription factors that can serve as biomarkers for regulation of BCoV pathogenesis in cattle, both for testing and treatment. A total of 11 genes were identified as significantly expressed during BCoV infection through literature searches and functional analyses. Eleven transcription factors were predicted to target those genes (AREB6, YY1, LMO2, C-Rel, NKX2-5, E47, RORAlpha1, HLF, E4BP4, ARNT, CREB). Function, network, and phylogenetic analyses established the significance of many transcription factors within the immune response. This study establishes new information on the transcription factors and genes related to host-pathogen interactome in BCoV infection, particularly transcription factors YY1, AREB6, LMO2, and NKX2, which appear to have strong potential as diagnostic markers, and YY1 as a potential target for drug therapies.

## 1. Introduction

Coronaviruses are enveloped RNA virus, spherical to pleomorphic in shape, with 20 nm spikes on the envelope. A member of the coronavirus family, within the beta A subtype [1,2], Bovine coronavirus (BCoV), is a respiratory and enteric pathogen that infects cattle and wild ruminant species. Like several other variations of coronaviruses, BCoV has additional 5 nm secondary spikes [2,3]. The larger spike interacts with sialic acid within receptors on the target cell, while the smaller spike contains hemagglutinin-esterase (HE), another protein critical in attachment to target cells [1,4]. The virus results in three distinct symptoms: calf diarrhea, winter dysentery with hemorrhagic diarrhea in adults, and respiratory infection in cattle of all ages [4,5]. Neonatal calf diarrhea (NCD) is the major cause of death in unweaned heifers [6,7]. These symptoms are devastating for the agricultural industry, with impacts including the cost of treatment and prophylaxis, increased vulnerability to additional diseases, increased mortality rates, long-term lowered growth rates, and lowered milk production [8,9]. Widespread BCoV infections can have disastrous effects, especially in countries like Uruguay or Vietnam, where cattle production is a primary source of income and the overall BCoV detection rate is 7.8% [7] and 33.3% [1], respectively.

Current diagnostic method involves sampling feces and intestinal contents and performing reverse transcription-polymerase chain reactions (RT-PCR) to assess for BCoV [7,8,9]. There are vaccines on the market for NCD and specifically BCoV, however, they are typically presented to pregnant cows rather than the calves [10,11,12]. Due to the difficulty in establishing a marker for targeted treatment of BCoV infections, recent developments within the field of transcription therapy might enable better diagnostic methods and finely targeted treatments. The traditional strategy for treatments is to block the “identified aberrant biochemical activity” [13]. However, with new developments in disease gene expression, an alternative idea is to target the transcription behind the undesired activity. Transcription factors (TFs) are proteins that bind to DNA to activate or inhibit transcription. Studies have shown success with targeting the DNA binding domain and successfully inhibiting TFs in vitro, including the identification of specific inhibitors for the TFs in human CREB, STAT3, and SOX2 genes [14,15,16].

Breakthroughs in computational and analytical tools have enabled complex research powered by preexisting database results [17]. Literature searches on databases such as PubMed or Google Scholar enable the identification of previously completed research and retrieval of genomic data that can be reanalyzed to serve new purposes, such as significantly expressed genes during BCoV infection [18]. Function analysis tools such as Gene Ontology [19] enable complex pathway analysis based off previous research. In part with the newly developed strategies to target transcription, transcription factor binding site prediction takes previously sequenced data with complex algorithms and extensive databases to produce a highly accurate list of transcription factors predicted to be involved with desired genes [20,21,22]. Finally, alignment software and phylogenetic tree developers such as MEGA enable analysis of evolutionary conservation between various species for specific sequences.

Although transcription targeting has had many successful studies utilizing different methods, few drugs have incorporated these methods so far [12]. Bovine coronavirus is comparatively under researched compared to other coronaviruses such as SARS-CoV, and many of the publicly available databases only feature few species, which typically does not include cattle. On the biomedical science repository PubMed, a search with the keyword “BCoV” return 166 results, while that with “SARS CoV” returns 12,793 results. Although SARS-CoV is a more commonly known disease, the outcome of BCoV infection is extensive and widespread. For any potentially economically devastating virus, specific and highly targeted drugs are necessary. With new breakthroughs potentially attainable by combing available datasets in databases and possibility for drug targeting, transcription factor targeted treatments for BCoV would provide protection against economically devastating outbreaks. Therefore, the goal of this study is to deploy up-to-date and modern computational tools to identify immune response genes and transcriptional regulatory elements during BCoV infection with a goal to pin-point candidate regulatory elements as drug targets.

## 2. Materials and Methods

### 2.1. Identification of Genes Associated with Bovine Coronavirus Pathway

This study utilized a data survey of genes significantly expressed during cattle infection with bovine coronavirus. The search was initially performed on PubMed (pubmed.gov) and later expanded to Google Scholar. An initial list of 77 genes was identified, with the majority from Aich et al. [23], and additional supporting literature. The list of genes was run through Gene Ontology [24,25], and important genes were recorded from significant pathways. GO terms with adjusted *p*-value less than 0.0001 and fold enrichment above 70 were considered significantly enriched from our gene list. This produced 31 different pathways, with 21 individual genes within them. Four genes were then removed from the list, as they were not in the original list compiled from literature. The remaining 17 genes were searched for their expression association with coronavirus in other species including human, murine, avian, feline, porcine, and canine. Seven of these 17 genes were removed because there were no supporting articles produced during the search, while gene NANS was added, due to its significance in the infection pathway of BCoV. In all, a total of 11 genes was catalogued and utilized for further analyses.

### 2.2. Prediction of Disease Associated Bovine Transcription Factors

The sequences and locations of the 11 genes were identified using NCBI nucleotide databases. The complete bovine nucleotide sequences along with accession numbers were retrieved and saved for analysis: TLR7 (NM_001033761), TLR9 (NM_183081.1), IRF1 (NM_001191261.2), IL-6 (NM_173923.2), CEBPD (NM_174267), TRAF2 (XM_005213525.4), RHOA (NM_176645.3), TP53 (NM_174201.2), CEBPB (NM_176788.1), SRC (NM_001110804.2), and NANS (NM_001046482.1). These genes were then used as seeds to identify transcription factors through different transcription factors prediction software. To increase accuracy, three computational tools were utilized; AnimalTFDB [22] and GeneXplain [26] that uses Match and P-Match algorithms for gene regulation. AnimalTFDB was run using the default settings, but both Match and P-Match algorithms on GeneXplain were adjusted to use the cutoff selection “0.95 and 1.0 as mat. sim. and core sim.” With Venny [27], TFs that appeared in the results of at least two software were considered significant for further analysis. We then searched for transcription factors that target multiple genes. This step allowed us to identify significant transcription factors that were substantially targeting multiple genes across the entire list of genes, to limit false positives and increase predicted relation to BCoV infection. After these steps were performed, only 11 TFs remained, which were then included for further analyses.

### 2.3. Functional Analysis of Transcription Factors and Gene Targets

Using Gene Ontology [24,25], we performed functional analysis of the 11 transcription factors and 11 genes previously identified as significant in BCoV infections. Pathways with *p* < 0.001; False Discovery Rate (FDR) < 0.05 and a fold enrichment value over 70 were recorded, along with the genes and transcription factors involved with them were considered important. Immune specific pathways were highlighted for later analysis.

### 2.4. Network Analysis of Transcription Factors and Genes

Using STRING database [28], we performed a network analyses of TFs, genes, and the TFs and gene. The software produces a network of the genes and TFs through colored connections representing established protein-protein associations. To develop a network through the tool Cytoscape (v3.8.0, [29], the identified TFs and their target genes, along with the pathways from the GO functional annotation, were compiled and used as input into the software.

### 2.5. Phylogenetic Analysis of Conserved Transcription Factors

To determine conservation of the identified transcription factors, sequences were retrieved from the GenBank database and run through BLAST. The topmost 25 hits were selected for different species based on the E-values from the BLAST search, then run through the MEGA (v7) software for multiple sequence alignment. Phylogenetic trees were then developed using the Neighbor-Joining Tree method [30].

## 3. Results

### 3.1. Identification of Genes and TFs Associated with Bovine Coronavirus

**Figure 1:**
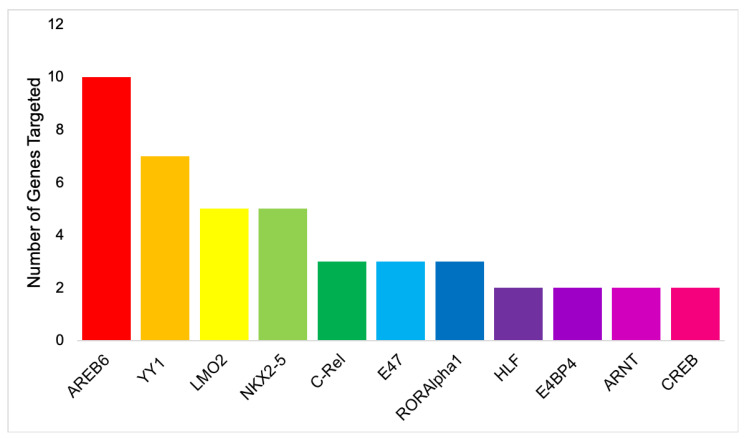
Bar chart illustrating the number of genes identified as targets for each transcription factor.

Literature searches and pathway analyses identified a total of 10 genes that appeared important in BCoV infections, as illustrated in Table 1. As explained in the Methods section, genes were recorded if they appeared in at least one significant pathway, with significance relying on the fold enrichment and the *p*-value. The gene NANS was added due to its significance in BCoV infection in the host, bringing the total of genes to 11. NANS is the gene responsible for producing N-acetyl 9-O-acetylneuraminic acid, the sialic acid within the host receptor BCoV utilizes to bind to host cell. From the binding site predictions, 11 transcription factors were identified. All factors were identified by at least two unique software in at least two different BCoV identified genes, with some identified in up to 10 different genes, as shown in Table 2 and Table 3. The only TF to appear in all three prediction software was NKX2-5. For three genes, NKX2-5 appeared in the results of two software, and for two genes, it appeared in all three. AREB6, YY1, and LMO2 all also appeared frequently in the predictions. As illustrated in Figure 1 and Table 3, most TFs were identified for two or three genes, which emphasizes a relationship between the genes, however, not necessarily a relationship with the factors used to establish the greater list of genes. However, with transcription factors that appear in 5, 7, or 10 of the 11 identified genes (NKX2-5, LMO2, YY1, and AREB6), there appears to be a potential relationship with the defining factors of the original gene list.

### 3.2. Functional Analysis of Transcription Factors and Genes 

**Figure 2:**
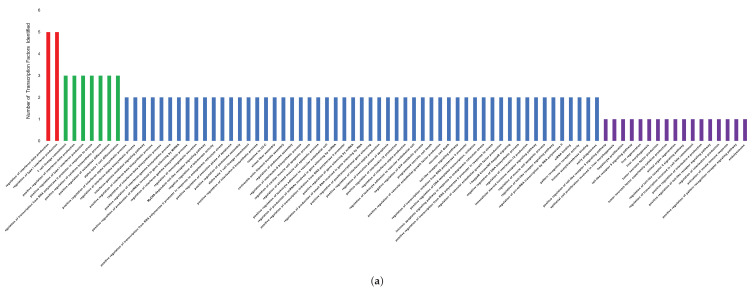

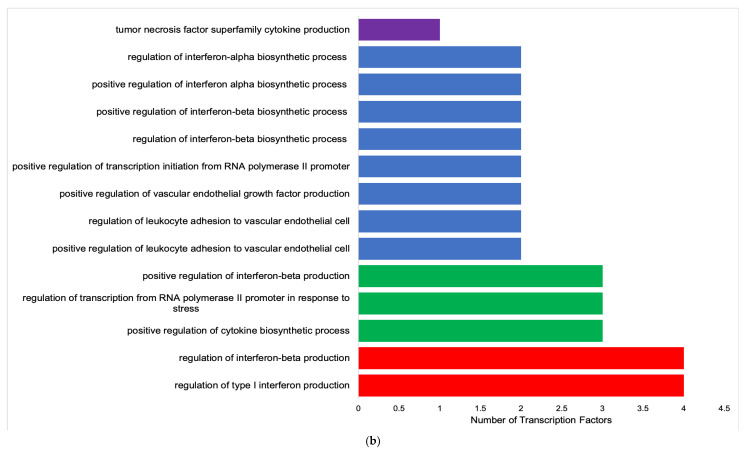
Pathway analysis of identified target genes and transcription factors; (**a**) Bar chart showing the number of genes or transcription factors identified for each significant pathway. Pathways with a red bar identified five genes and/or transcription factors, pathways with a green bar identified three, pathways with a blue bar identified two, and pathways with a purple bar identified one gene or transcription factor. (**b**) Bar chart showing the number of genes and transcription factors identified for each immune specific pathway. Immune specific pathways with a red bar had four genes and/or transcription factors, pathways with a green bar had three, pathways with a blue bar had two, and pathways with a purple bar had one.

After a list of genes and transcription factors were identified, functional analysis was performed to identify significant pathways. A pathway was considered significant if the fold enrichment was over 70 and *p*-values less than 0.00041. Figure 2a represents the number of genes and transcription factors identified within each pathway. The majority of the pathways only reported a total of two genes or transcription factors. However, the pathways “regulation of interferon-β production” and “regulation of type II interferon production” identified the same four genes and transcription factors: TLR7, YY1, TLR9, and IRF1. Figure 2b also represents results from the function analysis, however, with specific focus on immune specific pathways. These pathways reported between one and four genes and transcription factors. They are shown in the Cytoscape network illustrated in the section below (Section 3.3), where they are integrated into a network of genes and transcription factors.

### 3.3. Network Analysis of Transcription Factors, Genes, and Genes and Transcription Factors

**Figure 3:**
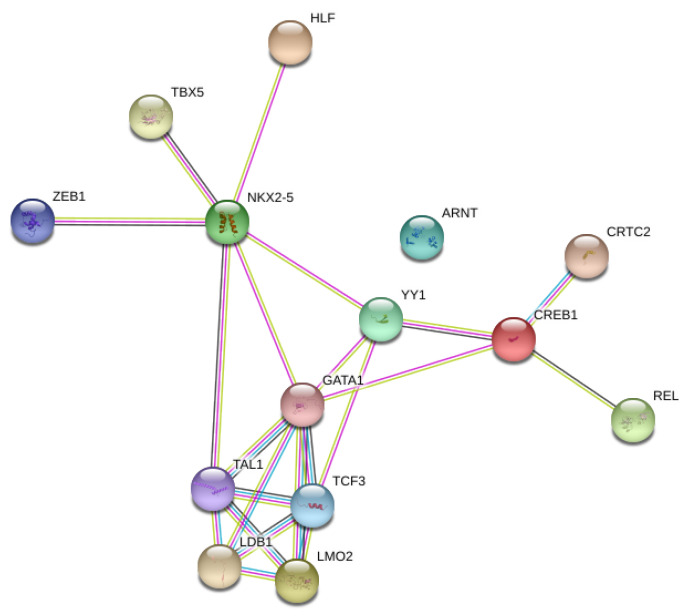
Network analysis of predicted transcription factors. ZEB1 is an alternative name for the transcription factor AREB6.

**Figure 4:**
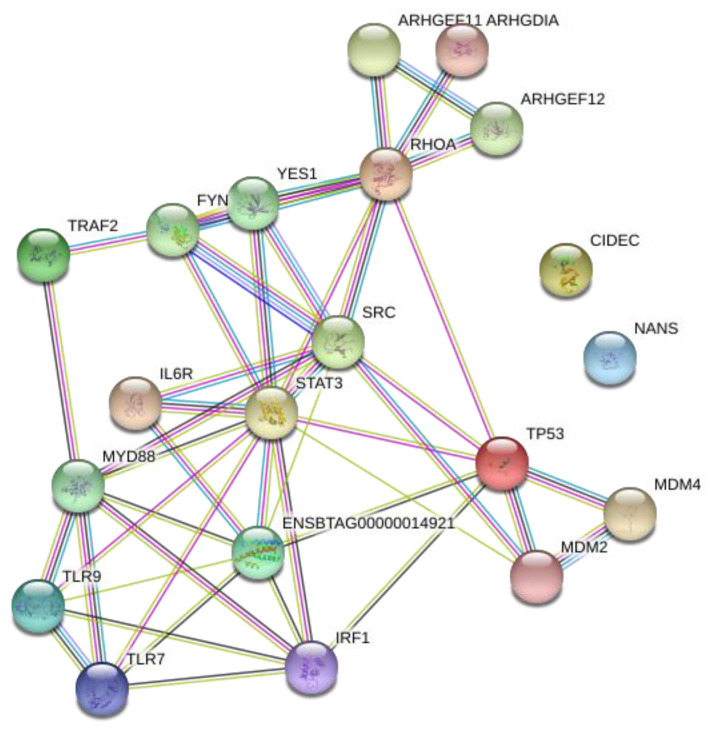
Network analysis of predicted genes. Protein-protein interaction network of immune genes associated with BCoV. (Edge color legend; blue: from curated database, pink: experimentally determined, green: text mining, brown: co-expression).

**Figure 5:**
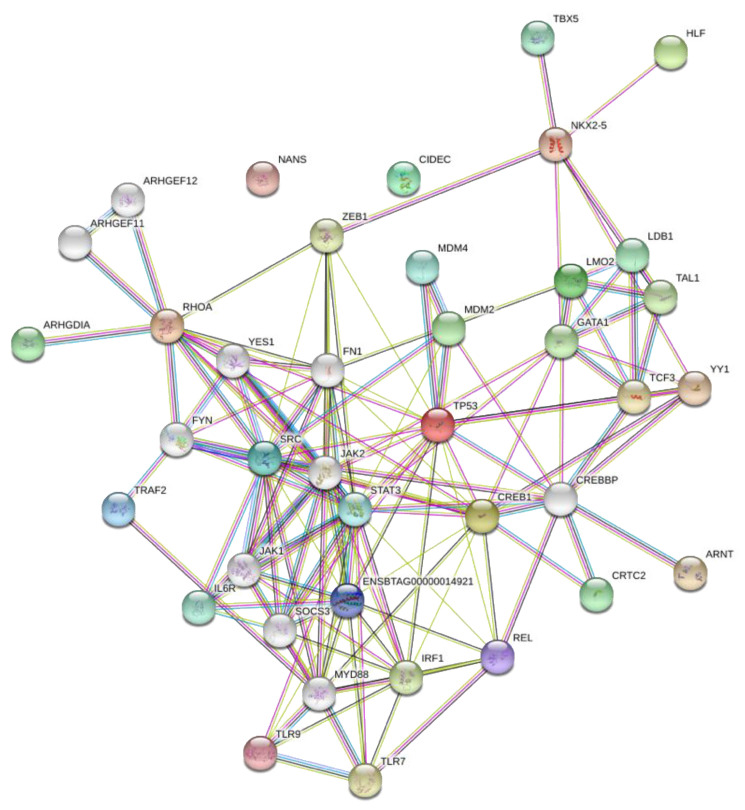
Network analysis of predicted genes and transcription factors. ZEB1 is an alternative name for transcription factor AREB6. (Edge color legend; blue: from curated database, pink: experimentally determined, green: textmining, brown: co-expression).

**Figure 6:**
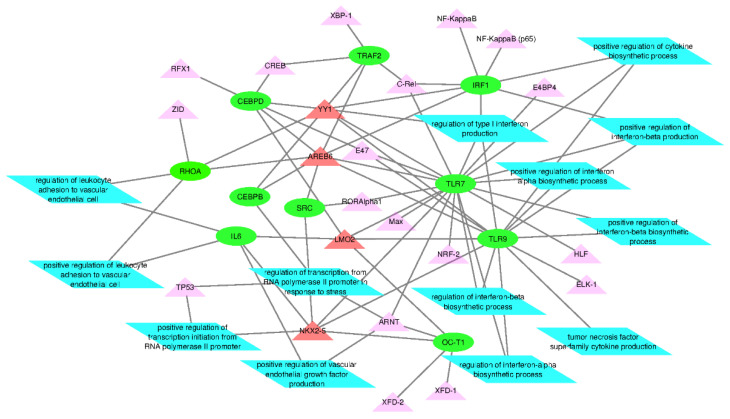
Network analysis of predicted genes, transcription factors, and immune specific pathways. The green circles represent genes and the blue parallelograms represent immune specific pathways. The transcription factors are represented by triangles, with the red representing many network connections, and the pink representing fewer network connections.

Network analysis of the relationships between the TFs, the genes, and then the genes and TFs were performed initially with STRING (Figure 3 and Figure 4). Since these figures are second or third interaction networks, there are genes included that are not included in original literature searches and pathway analyses and are not a part of the analysis. As shown by Figure 3, YY1 and NKX2-5 appear to be the largest hubs of predicted TFs. The TF ZEB1 is an alternative name of AREB6 and is only incorporated into the network through NKX2-5, which appears surprising. AREB6 is predicted in all genes except one, and therefore, is predicted for the same genes as the majority of the other transcription factors; however, the network only shows interaction with NKX2-5. Another surprising result from the network was that even in a second interaction network, ARNT does not have any interactions, which might represent an unrelated function to the other TFs. In Figure 4, some of the larger hubs from the original list of genes appear to be TLR9, TLR7, IL-6, TP53, and SRC, with the largest hub being RHOA. NANS is unconnected in this network, which is most likely due to its specific function as a gene. In Figure 5, a third interaction relationship, NANS, is still unconnected. ARNT becomes attached through an additional gene to YY1 and TP53, along with many other additional genes. NKX2-5 appears to be less of a hub than other transcription factors like ZEB1 (AREB6) and LMO2. The genes have much larger hubs than the transcription factors, with SRC, RHOA, and TP53 illustrating some of the largest original hubs. Figure 6 depicts the Cytoscape network, which incorporates transcription factors, genes, and immune significant pathways. Similar to earlier STRING networks, TLR7 appears to be a major hub of the network, creating the link between many transcription factors and pathways. YY1 appears to serve as a major transcription factor hub, even directly interacting with two major pathways “regulation of type I interferon production” and “regulation of interferon-β production”. Many of the transcription factors such as HLF, E4BP4, NRF-2, and ELK-1, only connect to a singular gene. Although HLF’s singular connection is to TLR7, the most prominent hub within the network, the transcription factors YY1 or AREB6, which interact with many genes including TLR7, appear to be a stronger target. 

### 3.4. Evolutionary Conservation of Transcription Factors

**Figure 7:**
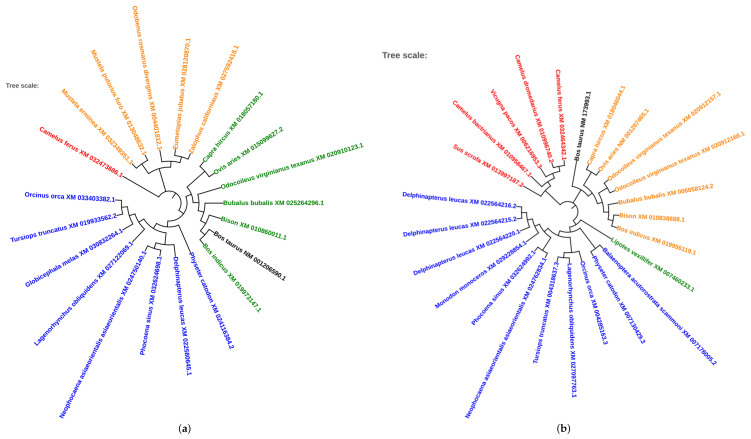

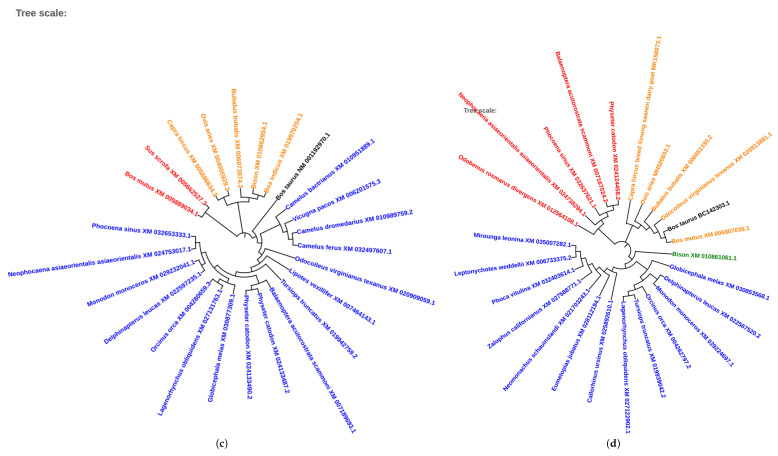

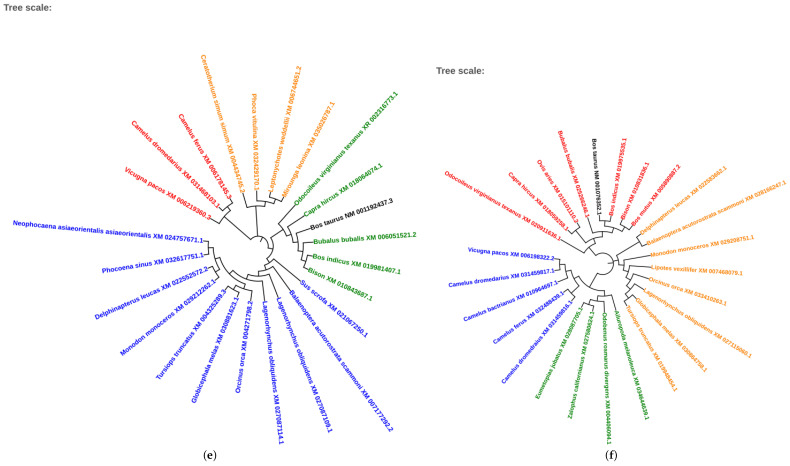

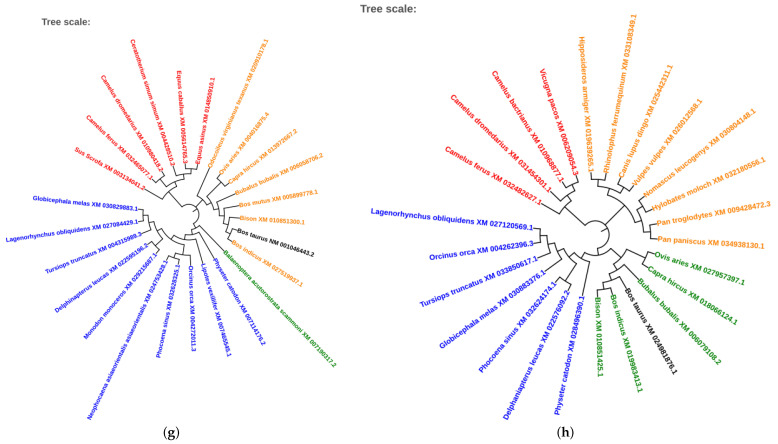
Phylogenetic tree representing the evolutionary conservation of transcription factors. *Bos taurus* is illustrated in black, and the nodes are color coded in four different groups, with red and orange representing the two different sub clades from the first divergence, and blue and green representing the other two sub clades. (**a**) Transcription factor AREB6; (**b**) ARNT; (**c**) C-Rel; (**d**) CREB; (**e**) HLF; (**f**) LMO2; (**g**) NKX2-5; (**h**) YY1.

Based on the results of TF prediction, homolog searches were performed through NCBI BLAST to retrieve multiple sequences from unique species. Phylogenetic trees were produced for eight of the predicted transcription factors. The factors E47, RORAlpha1, and E4BP4 were not present on NCBI, Ensembl, or UniProt, and therefore were excluded from analysis. These trees are illustrated in Figure 7a–h. AREB6, which is depicted in Figure 7a, is highly conserved in comparison to *Bos taurus* within Bison and *Bubalus bubalis* clade. *Bos indicus* and *Odocoileus virginianus texanus* are also within the clade. ARNT, illustrated in Figure 7b, has less evolutionary conservation in relation to *Bos taurus*. The closest relation appears to be with *Ovis aries*. In Figure 7c, which depicts the transcription factor C-Rel, there appears to be a lack of evolutionary conservation. C-Rel appears to be the closest to *Bos taurus* for *Capra hircus* and *Ovis aries*. The transcription factor HLF, present in Figure 7d, appears to be the most conserved in relation to *Bos taurus* in the species *Bubalus bubalis* and *Bos indicus*. *Odocoileus viriginianus texanus*, *Capra hircus* and Bison are also within the clade. Figure 7e illustrates the phylogenetic tree of CREB, which appears to have the most conservation from *Bos taurus* in *Bos mutus*. LMO2, the transcription factor for Figure 7f, is highly conserved between *Bos taurus* and *Bos indicus*. Bison and *Bos mutus* also appeared to be conserved in relation to *Bos taurus*. NKX2-5, one of the most predicted transcription factors, is illustrated in Figure 7g. *Bos indicus* appears to be very highly conserved in relation to *Bos taurus*, with *Bos mutus* and Bison also appearing closely conserved. Figure 7h depicts YY1, where *Bos indicus* and Bison appear to be the most conserved in relation to *Bos taurus*. Species within these phylogenies were mostly consistent between the trees, resulting in very similar phylogenetic trees and therefore very similar relationships of conservation. From these eight transcription factors, the species that appear to be highly conserved in relation to *Bos taurus* include *Bos mutus*, *Bos indicus*, *Bison*, and *Bubalus bubalis*.

## 4. Discussion

As one of the major risks to the cattle industry, bovine coronavirus outbreaks have the potential to cause significant economic effects and negative downturn in agricultural output. Causing both respiratory infections and weight-loss inducing diarrhea, along with long lasting lowered growth rates and diminished milk production, widespread BCoV infection would be devastating to countries with cattle as a primary source of GDP [8,9,31]. Despite the risk associated with uncontrolled outbreaks, BCoV is under researched and lacking in strong testing and treatment options. With recent progress in data mining, transcription factor prediction, and transcription targeted treatments, new testing and treatment methods are within reach. In this study, an extensive pipeline was developed to identify significant gene and transcription factor targets for future BCoV testing and treatment resources.

Through data mining and functional analyses, a list of 11 significant genes in BCoV infection were identified. Many of these genes were involved in immune response to infection, particularly inflammation. TLR7 and TLR9 are both implicated in pro-inflammatory signaling in response to recognized conserved viral molecular patterns and are key in the innate antiviral response [32,33]. TLR7 is activated by microbial derived nucleic acids while TLR9 is activated by dsDNA-derived CpG motifs [34]. TLRs are responsible for linking the innate and adaptive immune response, clearly a desired response to preserve or improve with a targeted drug. If a transcription factor was to repress TLR7 or TLR9, it would damage the immune response to BCoV. Other genes that would harm the desired immune response if repressed are RHOA, which is responsible for antigen presentation; T-cell activation; and chemokine, cytokine, and growth factor binding [35,36]; and IRF1, which is responsible for defense of host cells through the regulation of autoimmunity, inflammation, viral infections, and innate and adaptive immune responses [10]. The disruption of any of these genes will most likely affect the immune response. As seen from our results, the 10 transcription factors that target these four genes (HLF, YY1, AREB6, C-Rel, NKX2-5, E47, E4BP4, RORAlpha1, LMO2, ARNT) will either activate or repress the immune response through the activation or repression of these four genes.

IL-6 is also involved in immune reactions, however, as a pro-inflammatory cytokine, responsible for acute phase responses [37,38,39]. As pro-inflammatory cytokines result in inflammation, tissue damage, fever, and potential shock and even death [40], which are found to be increasingly expressed within respiratory tracts [41], a transcription factor enabled activation of the IL-6 gene is inferred to increase the potential of these negative effects. Similar to IL-6, NANS is inferred to be an undesirable target for transcription factor activation. The repression of the gene NANS would logically improve the desired immune response. NANS is the gene responsible for the production of N-acetyl 9-O-acetylneuraminic acid [42,43]; the sialic acid BCoV binds to on host cell receptors. The use of transcription factors to repress NANS would limit the production of N-acetyl 9-O-acetylneuraminic acid and reduce the opportunities for BCoV binding, thereby hampering the virus entry in to the host. Such limitations will reduce the capacity for infection, advance innate immunity and ameliorate further pathological outcome.

The other five genes lack a clear direct effect on the immune pathways and therefore have less inferable effects of potential regulation or activation through transcription factor targeting. CEBPB, involved in monocyte development [44], is key in the response to many types of infection but occasionally cause immunopathology [45]. TRAF2 is a key element of TNF [46,47], which has significant roles in inflammation. However, as also for CEBPD, SRC, and TP53, TNF has established links to cancer and its polymorphisms serving as susceptibility factor for many infectious diseases [48]. CEBPD is a tumor suppressor [49,50], which identifies it as a bad candidate for repression. However, it has been reported to enhance oncogenic pathways in different contexts [50]. TP53 has also been identified as a tumor suppressor gene [51] and SRC implicated in some cancers such as sarcoma [52]. As many of the transcription factors target both genes that should not be repressed and genes that should be repressed, transcription factors must be carefully chosen as target, with gene function in mind. Additionally, transcription factors such as NKX2-5, which have been proven to cause heart defects or disease when mutated or deleted [53,54], have external effects that must be carefully considered in the decision whether to target them for treatments. Transcription factors selected as biomarkers for targeting should be predicted for genes such as TLR7, TLR9, RHOA, IRF1, and IL6 since they are activated early in infection. These genes have functions directly related to immune response pathways and have the highest probability of expression during BCoV. The possibility to activate them for a robust innate immune response through TF-targeted therapy would be important in the treatment of BCoV infection [55,56]. Given the diverse functions of the 11 genes identified as significantly expressed during BCoV infection, and the high overlap between the genes for predicted transcription factors, the transcription factor selected to serve as the target for therapy must be selected with those functions in mind.

## 5. Conclusions

This study identifies specific transcription factors that could affect host response to bovine coronavirus infection, particularly within the immune response system. Eleven genes were identified as significantly expressed during infection, and an extensive pipeline was developed to predict and analyze potential transcription factors targeting those genes. Transcription factor involved with many genes and pathways were inferred to have a stronger potential effect on the host if targeted, due to their role in activating or repressing many genes. For example, the targeting of AREB6 would regulate 10 of the 11 identified genes, including TLR7, TLR9, IRF1, and many more. We established the predicted effect of these transcription factors based on the function of their gene interactions and associated pathways. In total, this study establishes relationships between bovine genes, transcription factors, and immune pathways. Given that the genes NANS and IL-6 were identified as having potentially negative effect on immune response, the regulation/activation of genes to improve host immune response to BCoV must be done through transcription factors capable of repressing either or both of them. The best transcription factors we identified as potential markers for host-pathogen interactome in BCoV infection include NKX2-5, AREB6, YY1, and LMO2. Given the criteria above and the fact that YY1 could target TLR7 and TLR9 signaling, type I and II interferon production/pathways, reported as effective for virus clearance during infection, if activated, establishes YY1 as the best TF with the best potential as a drug target for BCoV therapy.

## Figures and Tables

**Table 1:** List of significant genes in BCoV infection and the number of significant pathways they appeared in.

SN	Genes	Number of GO Pathways	GO Pathways Identified Within
1	TLR9	11	positive regulation of interferon-α, β and γ biosynthetic process; regulation of interferon-α, β and γ biosynthetic process; positive regulation of toll-like receptor 9 signaling pathway; toll-like receptor 9 signaling pathway; positive regulation of interleukin-8 biosynthetic process; tumor necrosis factor production
2	TLR7	8	positive regulation of interferon-α, β and γ biosynthetic process; regulation of interferon-α, β and γ biosynthetic process; positive regulation of interleukin-8 biosynthetic process; regulation of interferon-γ biosynthetic process
3	RHOA	4	apolipoprotein A-I mediated signaling pathway; stress fiber assembly; contractile actin filament bundle assembly; positive regulation of podosome assembly
4	CEBPD	3	epithelial cell proliferation involved in liver morphogenesis; liver regeneration; liver morphogenesis
5	SRC	3	stress fiber assembly; positive regulation of podosome assembly; contractile actin filament bundle assembly
6	IRF1	3	Interferon-γ-mediated signaling pathway; contractile actin filament bundle assembly; positive regulation of interleukin-12 biosynthetic process
7	IL-6	2	positive regulation of production of miRNAs involved in gene silencing by miRNA; regulation of interferon-γ biosynthetic process
8	TP53	2	interferon-γ-mediated signaling pathway; positive regulation of production of miRNAs involved in gene silencing by miRNA
9	CEBPB	2	epithelial cell proliferation involved in liver morphogenesis; hepatocyte proliferation
10	TRAF2	1	regulation of interferon-γ biosynthetic process
11	NANS	0	

Key: TLR9: Toll Like Receptor 9; TLR7: Toll Like Receptor 7; RHOA: Ras homolog family member A; CEBPD: CCAAT Enhancer Binding Protein Delta; SRC: SRC Proto-Oncogene, Non-Receptor Tyrosine Kinase; IRF1: Interferon Regulatory Factor 1; IL-6: Interleukin 6; TP53: Tumor Protein 53; CEBPB: CCAAT Enhancer Binding Protein beta; TRAF2: TNF Receptor Associated Factor 2; NANS: N-Acetylneuraminate Synthase.

**Table 2:** List of significantly expressed genes during bovine coronavirus infection and the number of predicted transcription factors.

S/No	Genes	Number of Significantly Predicted TFs	Significantly Predicted Transcription Factors
1	TLR7	10	HLF, YY1, AREB6, C-Rel, NKX2-5, E47, E4BP4, RORAlpha1, LMO2, ARNT
2	IRF1	6	YY1, C-Rel, AREB6, NKX2-5, LMO2, ARNT
3	TLR9	5	YY1, E47, AREB6, LMO2, NKX2-5
4	CEBPD	5	YY1, E47, AREb6, CREB, LMO2
5	TRAF2	4	YY1 AREB6, C-Rel, CREB
6	TP53	4	HLF, AREB6, RORAlpha1, E4BP4
7	SRC	3	NKX2-5, RORAlpha1, AREB6
8	IL-6	2	LMO2, NKX2-5
9	RHOA	2	YY1, AREB6
10	CEBPB	2	YY1, AREB6
11	NANS	1	AREB6

**Table 3:** List of predicted transcription factors targeting 11 genes significantly expressed during infection with bovine coronavirus.

S/No	Transcription Factor	Number of Gene Targets	Genes Targeted
1	AREB6	10	TLR7, TLR9, IRF1, CEBPD, TRAF2, RHOA, TP53, CEBPB, SRC, NANS
2	YY1	7	TLR7, TLR9, IRF1, CEBPD, TRAF2, RHOA, CEBPB
3	LMO2	5	TRL7, TLR9, IRF1, IL-6, CEBPD,
4	NKX2-5	5	IRF1, IL-6, TLR7, TLR9, SRC
5	C-Rel	3	TLR7, IRF1, TRAF2
6	E47	3	TLR7, TLR9, CEBPD
7	RORAlpha1	3	TLR7, TP53, SRC
8	HLF	2	TLR7, TP53
9	E4BP4	2	TLR7, TP53
10	ARNT	2	TLR7, IRF1
11	CREB	2	CEBPD, TRAF2

All transcription factors, except for NKX2-5, were found in 2 softwares. NKX2-5 was found 3 times in two softwares, and 2 times in all 3 softwares (AnimalTFDB, Match, and P-Match).
